# Transcriptomic Profiling Reveals Gene Expression Changes in Mouse Liver Tissue During Alveolar Echinococcosis

**DOI:** 10.3390/genes16070839

**Published:** 2025-07-18

**Authors:** Xiongying Zhang, Qing Zhang, Na Liu, Jia Liu, Huixia Cai, Cunzhe Zhao, Kemei Shi, Wen Lei, Wanli Ma, Shuai Guo, Wei Wang, Xiao Ma, Mei Wang

**Affiliations:** 1 Department of Laboratory Medicine, School of Medicine, Jiangsu University, 301 Xuefu Road, Zhenjiang 212013, China; zxiongying1991@163.com; 2 Qinghai Institute of Endemic Disease Prevention and Control, Xining 811602, China; qhdfbzq@163.com (Q.Z.); 15297017601@163.com (N.L.); 15500745709@163.com (J.L.); huixia_1107@163.com (H.C.); 15509711441@163.com (C.Z.); 18797339634@163.com (K.S.); qhdfblw@163.com (W.L.); lop741253613@live.com (W.M.); 18797363572@163.com (S.G.); wwqhxn@126.com (W.W.)

**Keywords:** alveolar echinococcosis, liver tissues, mouse model, RNA sequencing, differentially expressed genes (DEGs)

## Abstract

Background/Objectives: Alveolar echinococcosis (AE), caused by *Echinococcus multilocularis* larvae, poses a significant global health concern. Primarily affecting regions in the northern hemisphere, such as northwest China, which are vital for animal husbandry, it often results in severe hepatic impairment in the host. However, there remains a dearth of knowledge concerning changes in gene expression profiles during the progression of AE. In this study, we employed transcriptome sequencing (RNA sequencing, RNA-Seq) to detect alterations in gene expression profiles in the liver tissues of mice with AE. Our aims were to understand the transcriptome differences in the liver during *E. multilocularis* infection and to explore the molecular mechanisms underlying the early progression of this disease. Methods: We established a mouse model of AE by intraperitoneally injecting protoscoleces of *E. multilocularis*. All the inoculated mice were randomly divided into four groups. Liver tissues were collected at 6, 12, 19, and 25 weeks after inoculation. Paired non-infected mouse-derived liver tissues were used as controls, and transcriptome sequencing was carried out. Results: A total of 629 differentially expressed genes (DEGs) were identified. Among them, 370 genes were upregulated and 259 genes were downregulated. Moreover, Gene Ontology (GO) and Kyoto Encyclopedia of Genes and Genomes (KEGG) enrichment analyses indicated that these DEGs were significantly associated with immune system modulation, the cell cycle, and the fibrosis process during the pathological changes. Additionally, weighted gene co-expression network analysis (WGCNA) identified several genes, including *CCNA2*, *BIRC5*, *KIF2C*, *OTC*, *TLR2*, and *NCKAP1L*. These hub genes involved in immunoinflammatory processes may be related to *E. multilocularis* larvae infection. Conclusions: The findings of this research provide a theoretical foundation for a more in-depth understanding of the molecular mechanisms of AE. They offer valuable insights into the molecular mechanisms and potential key factors involved in the pathogenesis of this disease.

## 1. Introduction

Alveolar echinococcosis (AE), a parasitic disease instigated by the larvae of the tapeworm *E*. *multilocularis*, poses a substantial health risk, as it primarily affects the liver and lungs of both humans and animals [1]. The disease is characterized by a prolonged incubation period, typically spanning from 1 to 5 years, with the majority of cases occurring in individuals aged 20 to 40 years. Approximately 95% of primary lesions are situated in the liver, presenting with a proliferative growth pattern characterized by external budding and distant metastasis, closely resembling liver cancer and commonly referred to as “parasitic cancer” [2,3]. Given its long-standing and severe consequences, diagnosing AE often takes decades, and during this time, the prospects of survival or cure are quite slim [4]. *E. multilocularis* invades and proliferates aggressively within the hepatic tissues, perpetually generating new cysts and eroding adjacent structures [5]. As a result, liver damage and lesions frequently compromise hepatocyte function [6].

Transcriptomics, as a cutting-edge technology, has found wide-ranging applications in modern biological research. Currently, it is extensively employed in basic research on microorganisms and plants, clinical diagnostics, and drug development [7,8]. RNA sequencing (RNA-Seq) has been utilized in the biological analysis of AE infection in liver and peripheral blood. Mouse experimental models are highly relevant to the pathogenesis of AE in humans. This is because rodents are the natural intermediate hosts of *E. multilocularis*. Therefore, identifying the transcriptional responses associated with experimental AE can establish a crucial research basis for elucidating the pathogenesis of AE and proposing innovative intervention strategies.

In this study, we successfully developed a mouse model infected with AE to systematically examine the gene expression profiles in liver tissues. We employed RNA-Seq technology at specific time points to comprehensively capture the dynamic changes in gene expression. Following the RNA-Seq analysis, we carried out quantitative real-time polymerase chain reaction (qRT-PCR) as a rigorous validation step for the results from the RNA-Seq experiment. To gain profound insights into the biological functions of differentially expressed genes (DEGs) in the disease development process, we performed Gene Ontology (GO) and Kyoto Encyclopedia of Genes and Genomes (KEGG) enrichment analyses. These sophisticated bioinformatics tools allowed us to map the DEGs to specific biological processes, molecular functions, and signaling pathways, thus shedding light on the underlying biological mechanisms driving the progression of AE. Furthermore, we utilized weighted gene co-expression network analysis (WGCNA) to identify genes that potentially play crucial roles in the infection caused by *E. multilocularis* larvae and its temporal progression. By constructing gene co-expression networks, we aimed to uncover the complex interplay between genes and identify key regulatory nodes that drive the development of alveolar echinococcosis.

## 2. Materials and Methods

### 2.1. Parasites and Experimental Animals

The *E. multilocularis* larvae were obtained from long-clawed mole voles bred in our laboratory for research purposes. A total of 80 six-week-old specific pathogen-free (SPF)-grade female mice were purchased from Spiff (Beijing, China) Biotechnology Co., Ltd. To ensure ethical compliance, this study received approval from the Animal Ethics Committee of the Qinghai Institute for Endemic Disease Control and Prevention (Approval No.: QDB2021-0007). All animal experiments were conducted in strict accordance with the guidelines for animal welfare.

### 2.2. Establishment of Alveolar Echinococcosis Infected Mouse Model

Long-clawed mole voles exhibiting visibly enlarged abdomens due to *E. multilocularis* infection were euthanized via cervical dislocation. Multilocular cystic tissue, appearing transparent and clustered in the abdominal cavity, was extracted, minced, ground, filtered, washed, and centrifuged in PBS buffer. The obtained suspension was transferred into a sterile culture dish and carefully rinsed, and the protoscoleces were separated. Subsequently, the 0.1% methylene blue staining method was employed to assess their viability, ensuring that it exceeded 90%. The protoscoleces were further washed with PBS containing 800 U/mL penicillin–streptomycin and prepared as a suspension. A total of 80 clean-grade female Kunming mice, aged 6 weeks, were procured. These mice were allowed a one-week acclimation period in the experimental environment. After acclimation, they were randomly allocated into experimental and control groups. In the experimental group, each mouse was intraperitoneally injected with 0.3 mL of protoscolex suspension (approximately 1000 protoscoleces) using disposable sterile syringes. In contrast, control group mice received an equal volume of PBS buffer. The number of mice taken for analysis from each of the two groups at 4 time points was 32 (infected = 16, non-infected = 16). All mice were housed under standard conditions with free access to water and food.

### 2.3. Collection and HE Staining of Liver Tissue

At 6, 12, 19, and 25 weeks post-infection with protoscoleces, all experimental and control groups were euthanized via cervical dislocation and subsequently dissected. Liver tissue samples were collected from regions within 1–5 mm of macroscopically visible parasitic lesions, rinsed in a 10% EDTA solution, transferred into cryovials, flash-frozen in liquid nitrogen, and stored at −80 °C for downstream analyses.

In parallel, liver tissues were fixed in 4% formaldehyde solution, dehydrated, and embedded in paraffin for sectioning. Following hematoxylin-and-eosin (HE) staining, pathological changes in the liver tissues were assessed under light microscopy.

### 2.4. RNA-Seq

Liver samples (≈2 g) were collected from perilesional areas (1–5 mm from macroscopic lesions) and processed for RNA-Seq. The cDNA libraries were sequenced on the Illumina sequencing platform by Metware Biotechnology Co., Ltd. (Wuhan, China). Briefly, total RNA from the liver tissues was extracted using Trizol reagent. The quality of RNA was checked using an Agilent 2100 Bioanalyzer. RNA samples that passed the quality check were used to construct libraries using the NEBNext^®^ UltraTM RNA Library Prep Kit from Illumina. mRNA with poly(A) tails was enriched using oligo(dT) magnetic beads, followed by RNA fragmentation using NEB Fragmentation Buffer. cDNA was synthesized, purified, and quantified using qRT-PCR (library effective concentration > 2 nM) before library inspection. After passing the library inspection, different libraries were pooled based on the desired target data volume. Sequencing was performed on the Illumina platform to generate 150 bp paired-end raw data. The control and experimental groups at each specified time point (6, 12, 19, and 25 weeks) comprised four biological replicates, designated as follows: control1-(1–4), Treat1-(1–4), control2-(1–4), Treat2-(1–4), control3-(1–4), Treat3-(1–4), control4-(1–4), and Treat4-(1–4).

### 2.5. Differentially Expressed Gene (DEG) Analysis

The DESeq2 software package [9,10,11] was employed to conduct differential expression analysis between the various sample groups, aiming to identify a subset of DEGs between the two biological conditions. The false discovery rate (FDR) was computed using the Benjamini–Hochberg method to adjust the *p*-values of the hypothesis tests. Criteria for selecting DEGs were set as |log2Fold Change| ≥ 1 and FDR < 0.05.

### 2.6. Gene Ontology (GO) Annotation and KEGG Pathway Enrichment Analysis

With a significance threshold set at q < 0.05, TBtoolsp [12,13] software was utilized to conduct GO annotation and KEGG pathway enrichment analysis on the DEGs.

### 2.7. Weighted Coexpression Network Analysis (WGCNA)

The gene co-expression network was constructed using the R version 4.2.2 software WGCNA 1.71 package [14]. The gene expression table was imported into the R language package and partitioned into distinct modules through clustering. Correlation coefficients with the sample or sample properties were calculated using modular eigenvalues. The in-module connectivity of each gene was computed, with genes displaying high connectivity likely representing core genes with potentially significant functions. Protein–protein interaction (PPI) network analysis was performed in the STRING database, and the data were imported into Cytoscape (v3.9.1) software. Key genes were obtained by applying the degree algorithm of the Cytohubba plugin.

### 2.8. qRT-PCR Analysis

Tissue RNA was isolated using a Total RNA Extraction kit (Beijing Solaibao Biotechnology Co., Ltd., Beijing, China) and transcribed into cDNA utilizing a cDNA reverse transcription kit (Shanghai Yisheng Biotechnology Co., Ltd., Shanghai, China) according to the manufacturers’ protocols. The resultant cDNA was subsequently amplified using a qPCR SYBR Green Kit (Shanghai Yisheng Biotechnology Co., Ltd.) in a thermocycler (Bio-Rad Laboratories, Inc., Hercules, CA, USA). GAPDH was used as the internal reference, and the relative gene expression level was evaluated by the 2^−ΔΔCt^ method. The primer sequence information is shown in Table S1.

## 3. Results

### 3.1. Autopsies of Mice and HE Staining of Liver Tissues

Upon conducting autopsies, it was determined that at 6, 12, 19, and 25 weeks post-inoculation with the original head segment, the majority of mice exhibited hepatic alveolar echinococcosis, with infection rates of 10/12, 12/12, 12/12, and 12/12, respectively. At six weeks post-infection, the lesions were confined to the vicinity of the hepatic portal vein. By the twelfth week, the individual lesions had spread, resulting in damage to the diaphragmatic peritoneum. By the twenty-fifth week, over half of the mice (7/12) had experienced diaphragmatic metastasis.

**Figure 1:**
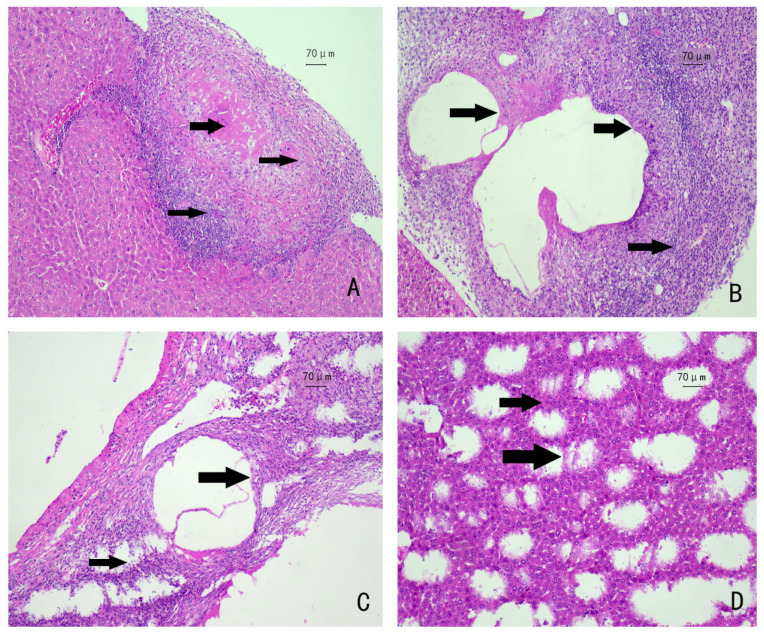
HE staining of mouse liver tissue: The arrows indicate the parasitic lesions in the livers of infected mice. (**A**) 6 weeks after infection with protoscolex; (**B**) 12 weeks after infection with protoscolex; (**C**) 19 weeks after infection with protoscolex; (**D**) 25 weeks after infection with protoscolex.

HE staining of tissue sections revealed that the liver tissue structure of mice in the blank control group was normal, with no obvious inflammatory cell infiltration observed in the portal area. Six weeks after *E. multilocularis* larvae infection in mice, hepatocyte edema and necrosis occurred around the lesion, and an inflammatory cell infiltration zone was visible, mainly consisting of macrophages and lymphocytes (Figure 1A). After 12 and 19 weeks of infection, fibrous cords formed, and a large number of inflammatory cells were seen in the lesion area. The inflammatory cells were densely and disorderedly arranged, gradually revealing hyperplastic fibrous connective tissue (Figure 1B,C). After 25 weeks of infection, honeycomb-like structures appeared in the livers of the mice, and the structure of liver lobules was severely damaged (Figure 1D).

### 3.2. Identification of DEGs in Liver Tissues at Various Stages of Hepatic Alveolar Hydatid Disease Development

In the experimental group’s livers, proliferating hepatocytes near the parasitic lesions were observed (A). Certain areas of coagulative necrosis were also observed (B, C). The arrows in the figures indicate the parasitic lesions in the livers of the infected mice. These lesions are characterized by the typical germinal layer and laminated layer of Echinococcus multilocularis, surrounded by a periparasitic cellular infiltration composed of macrophages, fibroblasts/myofibroblasts, and lymphocytes, spreading gradually from the center outward. The hepatocytes are disorganized, presenting a reticular structure (D).

A total of 32 samples, 16 from the experimental group and 16 from the control group, with 4 mice per timepoint, were subjected to transcriptome sequencing analysis. A robust dataset of 215.28 Gb of clean data was obtained, with each sample producing an effective data volume of 6 Gb, maintaining Q30 bases at a percentage of 92% or higher, and exhibiting an average GC content of 49%. Notably, the number of matched reads across all libraries exceeded 94%, confirming the high quality of sequencing and gene annotation, such that the data were suitable for subsequent bioinformatic analysis (Table S2).

**Figure 2:**
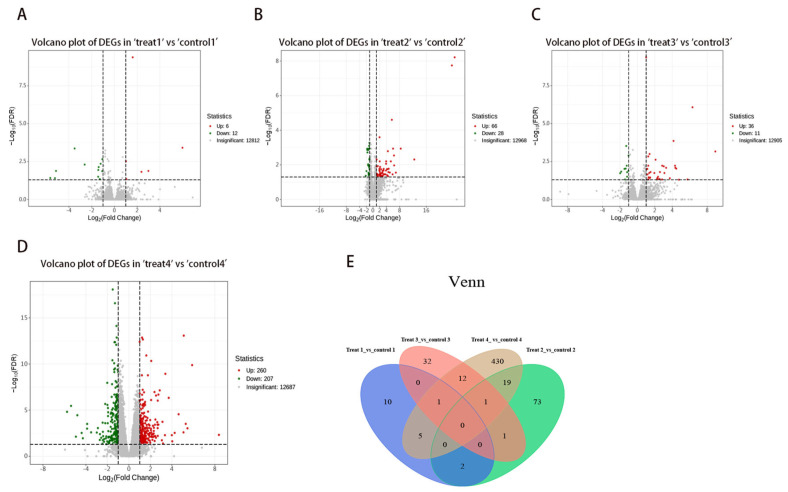
DEGs identification at different time points. (**A**–**D**) Volcano plots: (**A**) 6 weeks; (**B**) 12 weeks; (**C**) 19 weeks; (**D**) 25 weeks; (**E**) Venn diagram. Red represents upregulation, and green represents downregulation. The Venn diagram depicts the DEGs within each group and highlights the shared DEGs among these groups.

Differentially expressed genes (DEGs) were identified using stringent criteria, specifically |log2Fold Change| ≥ 1 and FDR < 0.05. Comparative transcriptomic analyses were conducted on liver tissues from mice at varying stages of *E. multilocularis* infection (Figure 2). A total of 629 differential genes were identified, with 370 showing upregulation, constituting 58.8% of the total differential genes, and 259 showing downregulation, accounting for 41.2% of the total differential genes. Notably, within the experimental group, 6 genes were upregulated and 12 genes were downregulated at 6 weeks post-inoculation (Figure 2A; Table S3); 68 genes were upregulated and 28 genes were downregulated at 12 weeks post-inoculation (Figure 2B; Table S4); 36 genes were upregulated and 11 genes were downregulated at 19 weeks post-inoculation (Figure 2C; Table S5); and 260 genes were upregulated, whereas 208 genes were downregulated at 25 weeks post-inoculation (Figure 2D; Table S6). Moreover, a Venn diagram depicting the differential expression profiles at the four timepoints reveals that 10, 73, 32, and 430 genes were specific to each timepoint, respectively (Figure 2E; Table S7).

### 3.3. Validation of Differential Genes by qRT-PCR

**Figure 3:**
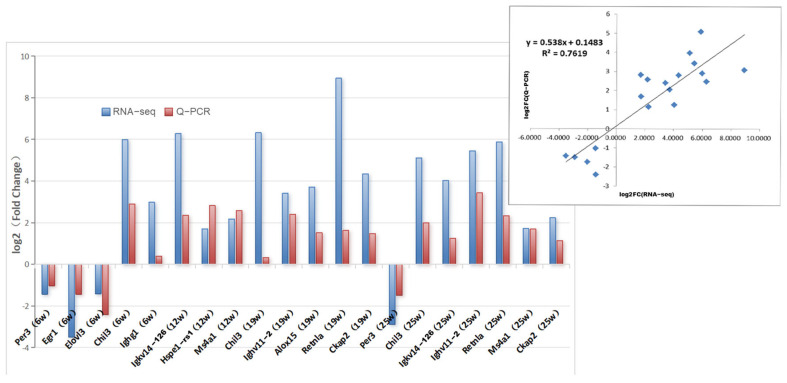
Validation of transcriptome sequencing data by qRT-PCR.

Twelve genes which showed differential expression at a minimum of two of the four experimental timepoints were selected for confirmation using qRT-PCR. The research findings indicate that the expression trends of these selected genes are consistent with those obtained from the RNA-Seq analysis. However, the performance of some genes in the RNA-Seq and qRT-PCR was not entirely identical, for example, that of *Ighg1* (at 6 weeks) and *Chil3* (at 19 weeks). Overall, the correlation coefficient was R2 = 0.7619 (*p* < 0.05). These findings highlight the reliability of transcriptome sequencing, as confirmed by the qRT-PCR analysis (Figure 3).

### 3.4. Function and Pathway Outcomes of GO Enrichment

GO enrichment analysis was conducted to investigate the relevant biological processes, cellular components, and molecular functions at various stages of infection progression (Figures S1–S3; Tables S8–S11). At the early stage (6 weeks), DEGs did not show enrichment in any biological processes, cellular components, or molecular functions. However, by the intermediate stage (after 12 weeks), DEGs exhibited enrichment in biological processes associated with antigen receptor-mediated signaling (GO:0050851), immune response-activating cell surface receptor signaling (GO:0002429), immune response-regulating cell surface receptor signaling (GO:0002768), and B cell receptor signaling pathways (GO:0050853). By 19 weeks, DEGs were enriched only in biological processes such as regulation of attachment of spindle microtubules to kinetochores (GO:0051988), response to stilbenoids (GO:0035634), mitotic sister chromatid segregation (GO:0000070), and mitotic nuclear division (GO:0140014). In the late stage (25 weeks), DEGs were associated with biological processes such as fat cell differentiation (GO:0045444), leukocyte aggregation (GO:0070486), regulation of chemokine production (GO:0032642), and response to topologically incorrect protein (GO:0035966). Overall, in the infected mice, distinct patterns of enriched functions emerged at different timepoints. By the 12-week mark, the predominant enriched functions were mainly associated with a wide array of immune and inflammatory responses. As time progressed to the 19th week, the focus of these functions shifted to the cell cycle process to regulate the cell proliferation state. Finally, by 25 weeks, clear manifestations of enriched functions were observed in the positive regulation of inflammatory responses and chemokine functions, as well as the negative regulation of anti-inflammatory factors and hydrolase activities.

### 3.5. Function and Pathway Results of KEGG Analysis

KEGG enrichment analysis was conducted to discern enriched signaling pathways across different timepoints during the progression of infection (Figures S4–S6; Tables S12–S15). At the initial stage, six weeks after inoculation, no significant enrichment of signaling pathways was detected. However, by the mid-stage, twelve weeks post-inoculation, DEGs were prominently found in the pathways related to protein digestion and absorption, as well as the B-cell receptor signaling pathway. Additionally, other signaling pathways, such as the Wnt signaling pathway, the MAPK (Mitogen-Activated Protein Kinase) signaling pathway, and the PI3K-Akt signaling pathway, were also involved. Subsequently, at 19 weeks post-inoculation, significant changes were observed in the DEGs associated with arachidonic acid metabolism and circadian rhythm regulation. Among them, the AMPK (AMP-activated protein kinase) signaling pathway was implicated. Notably, at 25 weeks post-inoculation, DEGs showed elevated expression levels in the pathways related to prion disease, the PPAR (peroxisome proliferator-activated receptor) signaling pathway, and the biosynthesis of unsaturated fatty acids.

### 3.6. Coexpression Network-Related Modules and Hub Genes

**Figure 4:**
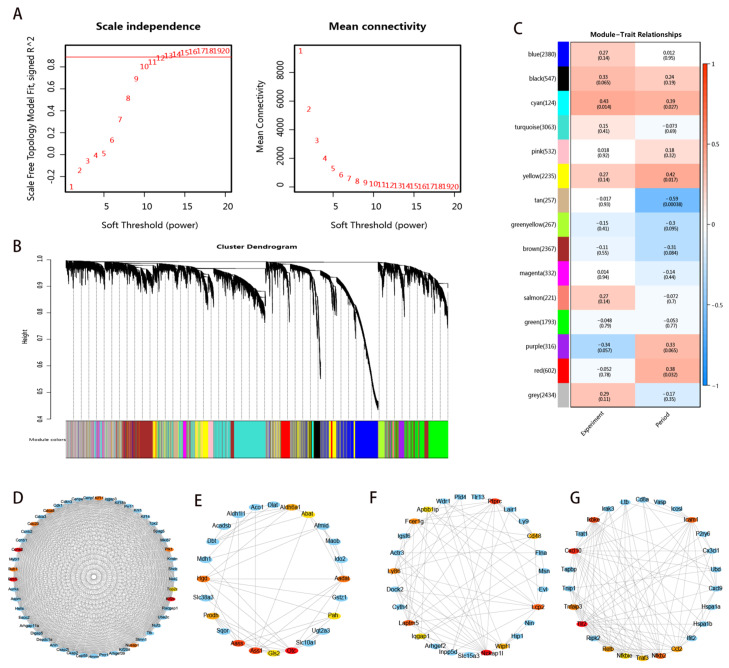
WGCNA analysis. (**A**) Determination of soft threshold of gene co-expression network. (**B**) Gene co-expression module hierarchical clustering map. (**C**) The correlation heatmap for *E. multilocularis* larvae infection and infection period. (**D**–**G**) Screening modules for gene co-expression networks and core genes: (**D**) cyan module; (**E**) tan module; (**F**) yellow module; (**G**) red module.

WGCNA was conducted on the transcriptome data to identify genes potentially linked to echinococcus infection. A total of 25,117 genes measured in the transcriptome data were analyzed. The co-expression threshold calculated by the software was set at 12 (Figure 4A). Subsequently, genes with similar expression patterns were clustered according to fragments per kilobase million (FPKM) values. The gene clustering tree generated from this process was then divided into distinct modules using the Dynamic Tree Cut method (Figure 4B). In total, fifteen different modules were identified, and each was represented by a unique color. The turquoise module had the largest number of genes, while the salmon module had the fewest. Pearson correlation coefficients were used to illustrate the relationships between the characteristic values of these modules and the trait data (Figure 4C). Remarkably, the cyan module showed positive correlations with both the infection condition (r = 0.43) and the infection period (r = 0.39). The yellow and red modules, however, were only positively associated with the infection period. On the contrary, the tan module showed a negative correlation (r = −0.59) with the infection period. The findings strongly imply that the DEGs within these modules could play a crucial role in Echinococcus infection. To identify the key genes, we selected the top 50 genes with the highest connectivity from the cyan, tan, yellow, and red modules. Among them, the core genes *CCNA2*, *BIRC5*, and *KIF2C* were obtained by the cyan module screening (Figure 4D); the core gene *OTC* was obtained by the tan module screening (Figure 4E); the core gene *TLR2* was obtained by the red module screening (Figure 4F); and the core gene *NCKAP1L* was obtained by the yellow module screening (Figure 4G).

## 4. Discussion

Currently, transcriptomic research on echinococcosis has advanced significantly, with extensive studies characterizing the hepatic transcriptional profiles and molecular regulatory networks involved in disease progression [15,16]. However, the intricate growth dynamics of AE and its underlying pathophysiological mechanisms remain incompletely elucidated. To concisely describe disease progression, we employed the staging criteria established by Zhang et al. [17], categorizing the infection timeline into early (before 60 days), intermediate (60 to 180 days), and late (after 180 days) stages. We elucidated the dynamic molecular alterations and associated signaling pathways that play critical roles in the early development of AE, providing novel insights into the pathogenesis and potential key factors of hepatic echinococcosis.

Microscopic examination of AE lesions demonstrates peripheral annular fibrous tissue hyperplasia, accompanied by infiltration of diverse immune cells—including lymphocytes, eosinophils, macrophages, and plasma cells—at the lesion margins. This immune-rich periphery constitutes an inflammatory microenvironment that shares histological similarities with hepatocellular carcinoma. AE lesions lack a complete fibrous capsule and instead exhibit invasive growth patterns, facilitated by a unique immune microenvironment that promotes parasite persistence and immune evasion [18]. Immune mechanisms play a decisive role in AE progression, governing whether the infection resolves spontaneously or establishes chronic persistence post-hepatic colonization, as observed in both human patients and animal models [19]. The long-term parasitic survival of larvae is primarily mediated by regulatory T cells (e.g., Th1 and Th2) and related cytokines (e.g., IL-10 and TGF-β) [20].

This study revealed that at 6 weeks post-infection, transcriptional profiling showed minimal differential gene expression and no significantly enriched pathways, suggesting an initial immune-tolerant phase. By 12 weeks, however, immune-related signaling pathways were markedly activated, with early Th1-mediated responses playing a protective role by suppressing parasite proliferation and mitigating granuloma-associated tissue damage [21,22]. As infection progressed to the middle-to-late stages, *E. multilocularis* larvae selectively stimulated the release of Th2-associated chemokines, suppressing pro-inflammatory cytokines [23]. This Th2 polarization promoted a humoral-dominant immune response, effectively dampening host protective immunity and facilitating parasite immune evasion and sustained pathogenicity [24]. Concurrently, leukocyte aggregation and chemokine production further recruited monocytes, lymphocytes, and other immune cells to the liver. On one hand, this promotes the proliferation, survival, and metastasis of *E. multilocularis* larvae. On the other hand, it leads to liver damage, chronic progression, and fibrosis [25].

KEGG enrichment analysis revealed that, after 12 weeks of *E. multilocularis* infection, multiple signaling pathways contribute to fibrosis progression, predominantly the Wnt, MAPK, PI3K-Akt, and AMPK signaling pathways. Previous studies [26,27] have established a critical role for the MAPK signaling pathway in mediating the inflammatory response and hepatic fibrosis following E. multilocularis infection. In both human AE and mouse models, MAPK pathway activation drives macrophage polarization toward an M2 phenotype [28], leading to the secretion of transforming growth factor-α (TGF-α) and TGF-β—key mediators that stimulate hepatic stellate cell (HSC) proliferation, transdifferentiation, and fibrogenesis [29]. Moreover, *E. multilocularis* protoscoleces modulate glycolysis via the PI3K/AKT/mTOR and AMPK pathways, further promoting M2 macrophage polarization and accelerating AE pathogenesis [30]. While local hepatic fibrosis initially serves a protective function—sequestering the parasite and limiting its intrahepatic dissemination—macrophage depletion impairs early larval clearance and suppresses fibrogenesis, paradoxically facilitating E. multilocularis proliferation [31].

Activation of the PPAR signaling pathway has been observed in advanced-stage disease. PPARs play crucial roles in adipocyte differentiation and lipid metabolism [32]. The PPAR family comprises three subtypes: PPARα, PPARγ, and PPARβ/δ, which exhibit divergent biological functions. PPARγ is known to exert anti-inflammatory effects [33], whereas PPARβ/δ promotes pro-inflammatory responses [34]. Interestingly, during tumorigenesis, PPARβ/δ may antagonize PPARγ’s activity [35]. Accumulating evidence suggests that PPARβ/δ exhibits strong tumor-promoting properties [36,37]. Moreover, studies indicate that downregulating PPAR pathway genes can mitigate inflammation by suppressing transcription factors and modulate the cell cycle during tumor progression by inducing apoptosis [38].

Through WGCNA, six core genes, namely, *CCNA2* (cyclin A2), *BIRC5* (baculoviral IAP repeat-containing 5), *KIF2C* (kinase family member 2C), *OTC* (ornithine transcarbamylase), *TLR2* (toll-like receptor 2), and *NCKAP1L* (NCK-associated protein 1-like), were identified. These genes are potentially implicated in cell cycle regulation and B-cell signal transduction. There is a significant correlation between the expression level of *CCNA2* and the infiltration of CD4^+^ T cells. It may play a role in regulating the remodeling of the immune microenvironment and the activation of macrophages [39], thereby increasing the degree of immune infiltration in AE. The expression of *BIRC5* is related to the activation of HSCs, which may promote the occurrence of liver fibrosis. *KIF2C*, a member of the kinesin family, participates in multiple biological processes, including tumor invasion, metastasis, immune escape, and cell senescence [40]. *NCKAP1L*, also known as HEM1 (Hematopoietic protein 1), serves as a key regulator of the signal intensity of the B-cell receptor. It is essential for the development and maintenance of B-cell homeostasis [41]. *TLR2* is well-known for its prominent biological function of promoting the synthesis and release of inflammatory factors. It can activate immune cells such as macrophages and dendritic cells. High expression of *TLR2* may regulate antigen presentation, parasite growth, and granuloma formation [42]. An in vivo study demonstrated that in patients with HAE, the increased expression of mRNAs like *TLR2* and the elevated levels of related cytokines (IFN-γ, IL-5, IL-23, and IL-10) can protect the parasites from the host’s immune system [43]. Additionally, some scholars have reported that inflammatory stimulation and high expression of *TLR2* can effectively modulate the tissue-invasive growth of *E. multilocularis* larvae and its persistence within the host [44,45].

In summary, during the infection of the host by *E. multilocularis* larvae, a complex process unfolds. The establishment of the hepatic immune microenvironment and the activation of multiple signaling pathways drive the development of alveolar echinococcosis fibrosis. This sets the stage for a dynamic interplay between immune–inflammatory and fibrogenic pathways. It dictates how *E. multilocularis* larvae manage to evade the host’s immune surveillance, promotes their invasive proliferation within the hepatic tissue, and ultimately determines their long-term survival within the host. In essence, the balance and interaction between immune–inflammatory and fibrotic pathways are the key factors shaping the outcomes of alveolar echinococcosis.

Our current research has shed light on the dynamic changes in genes and their associated signaling pathways during the progression of infection by E. multilocularis larvae. Nevertheless, substantial gaps in our knowledge still exist, and reliable experimental validation is urgently needed.

## Data Availability

The original contributions presented in this study are included in the article. Further inquiries can be directed to the corresponding authors.

## Supplementary Materials

The following supporting information can be downloaded at https://www.mdpi.com/article/10.3390/genes16070839/s1, Table S1: qRT-PCR primer sequence information; Table S2: Summary of transcriptome sequencing data results; Tables S3–S6: DEG identification at four time points (weeks 6, 12, 19, and 25); Table S7: Venn diagram at four time points; Tables S8–S11: GO enrichment analysis at four time points (weeks 6, 12, 19, and 25); Tables S12–S15: KEGG pathway enrichment analysis at four time points (weeks 6, 12, 19, and 25); Figures S1–S3: Bubble chart for GO enrichment analysis; Figures S4–S6: Bubble chart for KEGG enrichment analysis.

## Abbreviations

The following abbreviations are used in this manuscript:

AE	Alveolar echinococcosis
*AMPK*	AMP-activated protein kinase
*BIRC5*	Baculoviral IAP repeat-containing 5
*CCNA2*	Cyclin A2
DEGs	Differentially expressed genes
EDTA	Ethylene Diamine Tetraacetic Acid
FPKM	Fragments per kilobase million
GO	Gene Ontology
HEM1	Hematopoietic protein 1
HSCs	Hepatic stellate cells
KEGG	Kyoto Encyclopedia of Genes and Genomes
*KIF2C*	Kinase family member 2C
M2	Macrophages 2
MAPK	Mitogen-Activated Protein Kinase
*NCKAP1L*	NCK-associated protein1-like
*OTC*	Ornithine transcarbamylase
PPAR	Peroxisome proliferator-activated receptor
qRT-PCR	Quantitative real-time polymerase chain reaction
RNA-Seq	RNA sequencing
TGF-β	Transforming growth factor-beta
*TLR2*	Toll-like receptor 2
WGCNA	Weighted gene co-expression network analysis
