# Supplementary figures and images for: Transcriptomic Profiling Reveals Gene Expression Changes in Mouse Liver Tissue During Alveolar Echinococcosis
Source: Genes (Basel). 2025 Jul 18;16(7):839. doi: 10.3390/genes16070839 (PMC12294577; doi:10.3390/genes16070839)

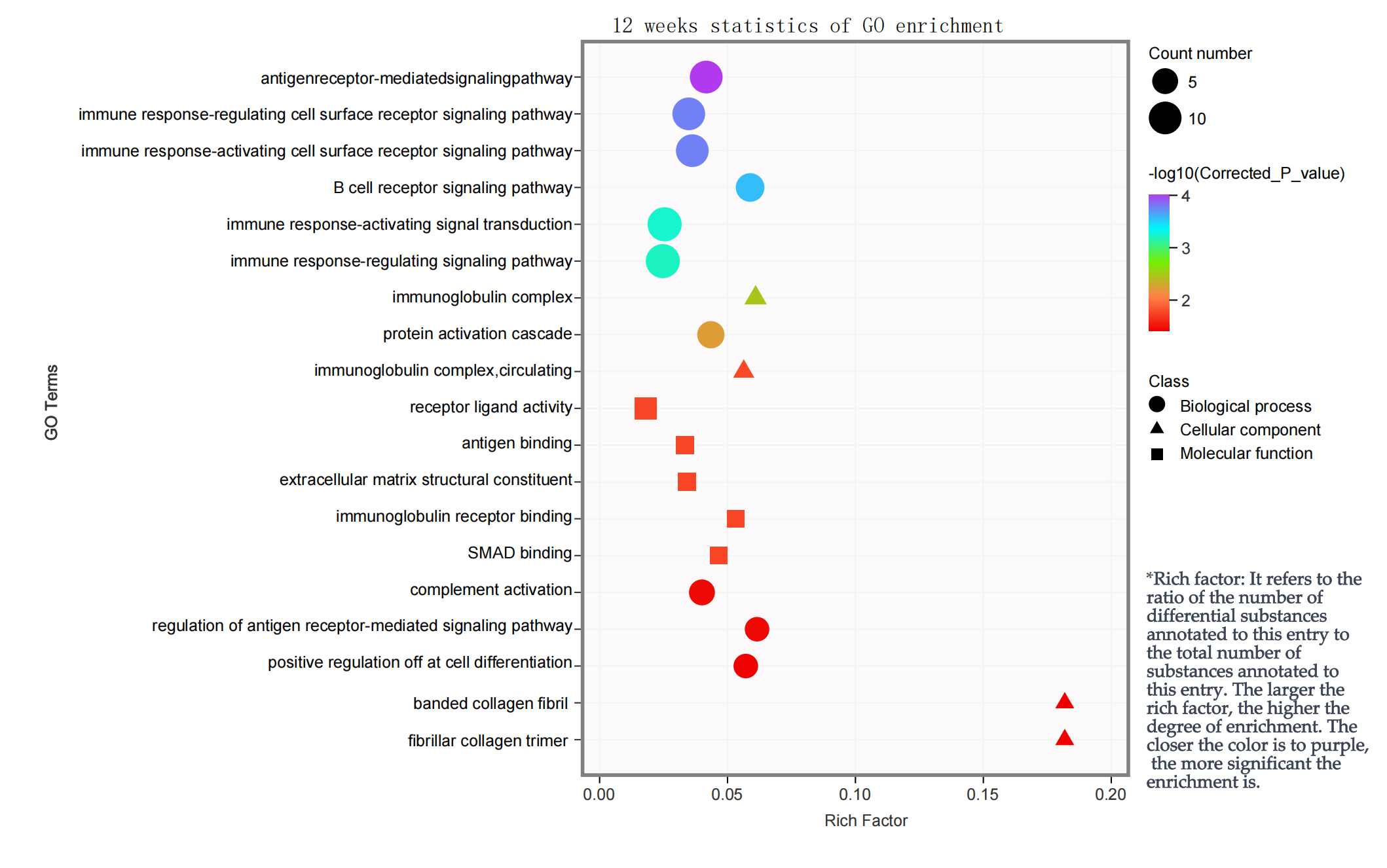

Supplement: Supplementary file 1 [file genes-16-00839-s001.zip › Figure S1.png]

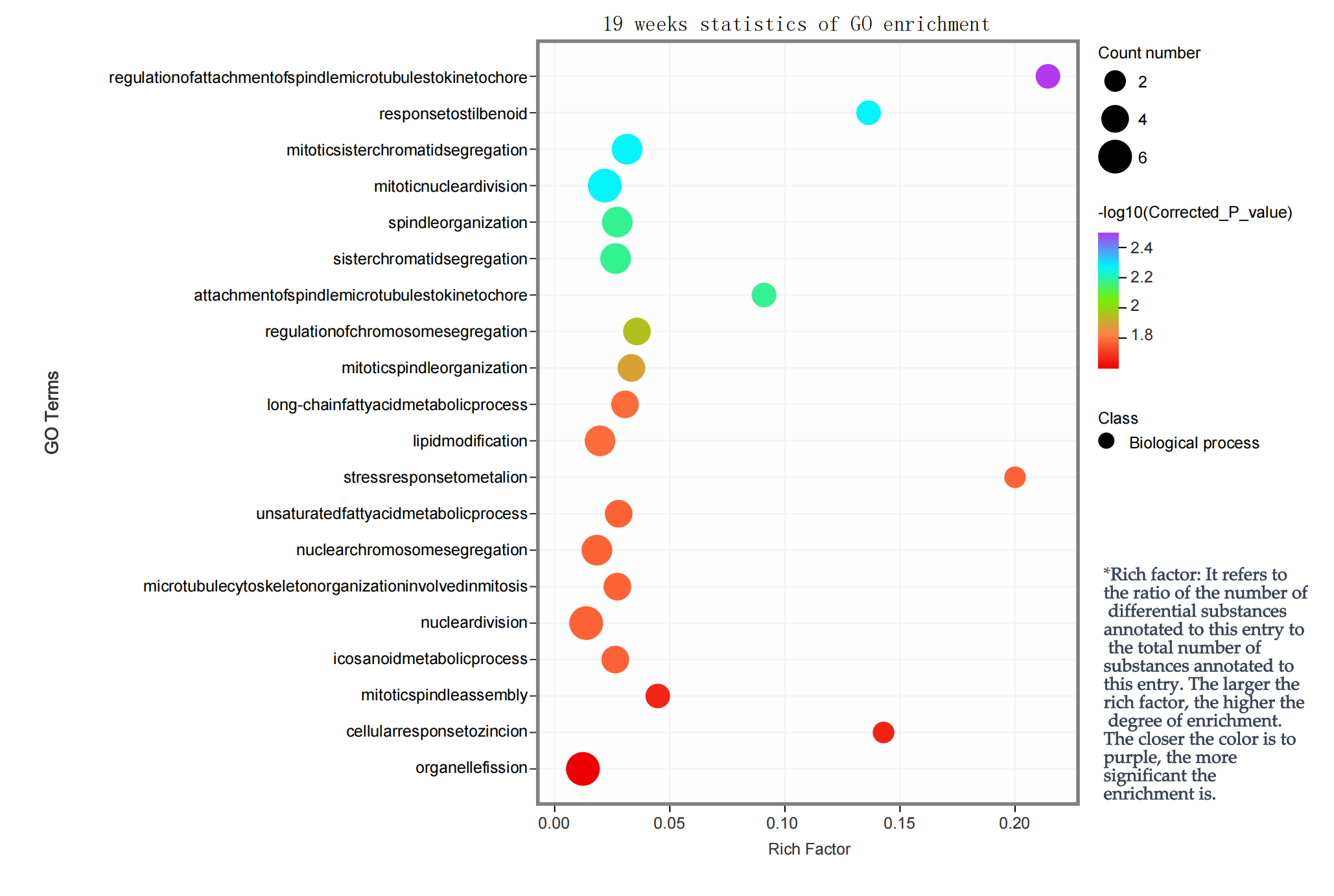

Supplement: Supplementary file 1 [file genes-16-00839-s001.zip › Figure S2.png]

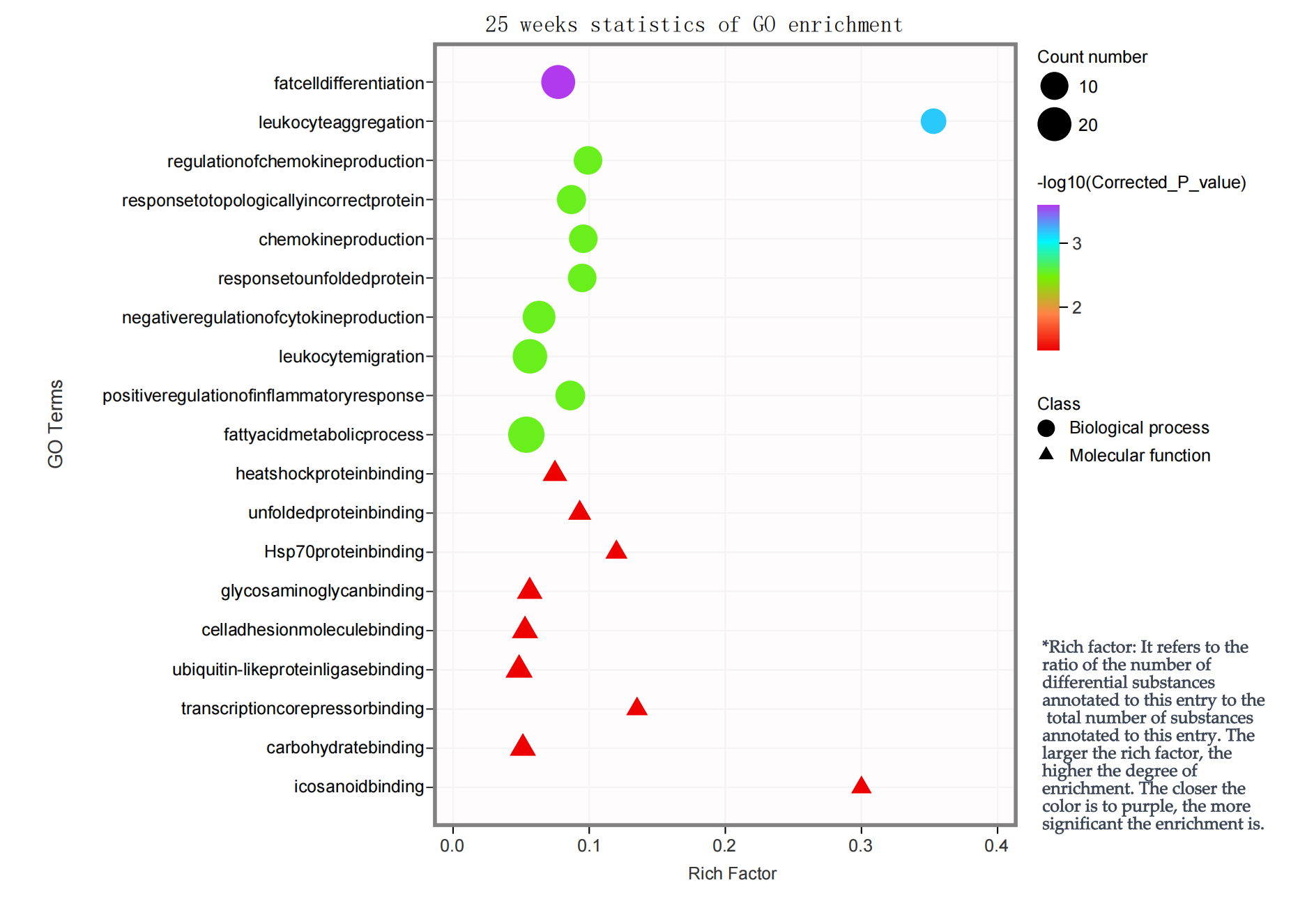

Supplement: Supplementary file 1 [file genes-16-00839-s001.zip › Figure S3.png]

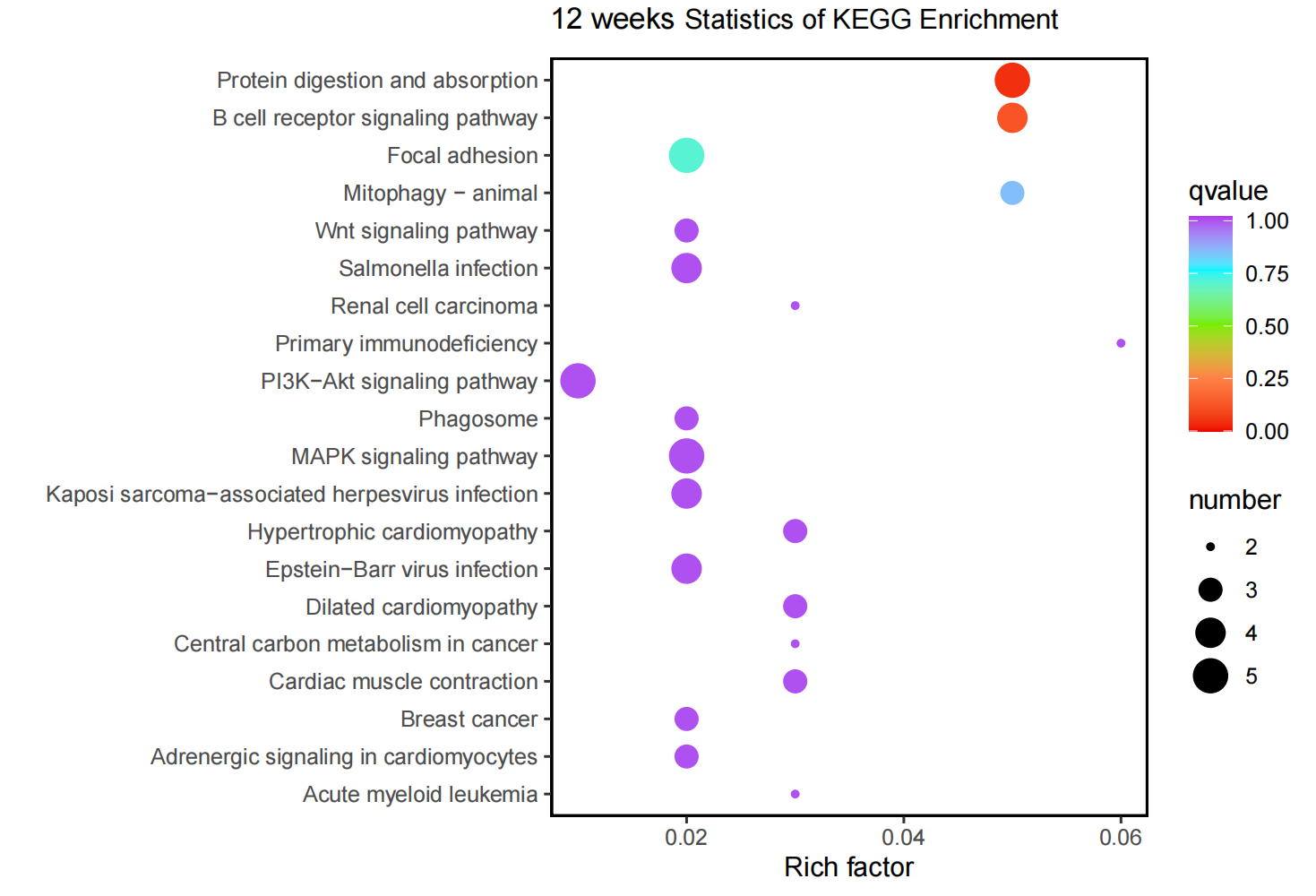

Supplement: Supplementary file 1 [file genes-16-00839-s001.zip › Figure S4.png]

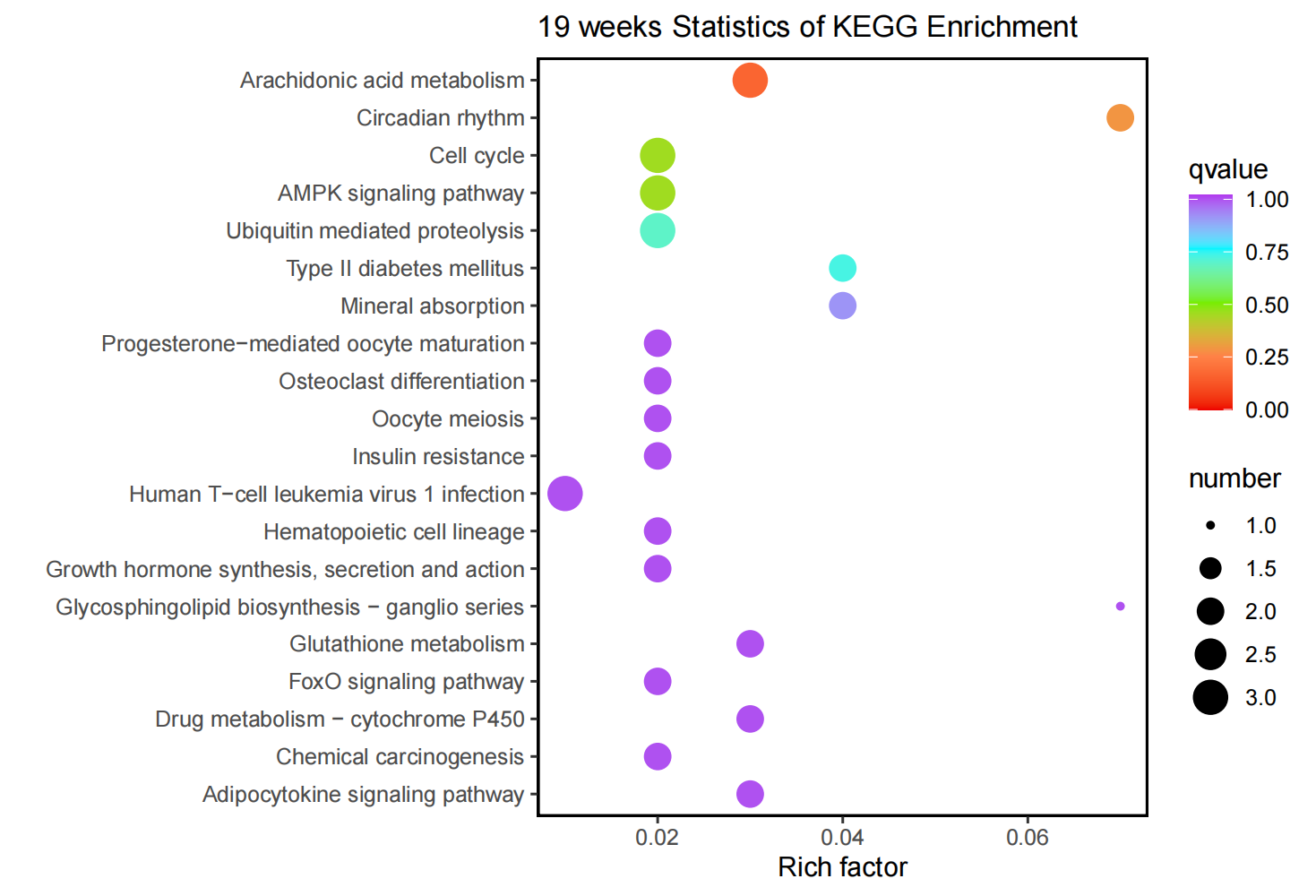

Supplement: Supplementary file 1 [file genes-16-00839-s001.zip › Figure S5.png]

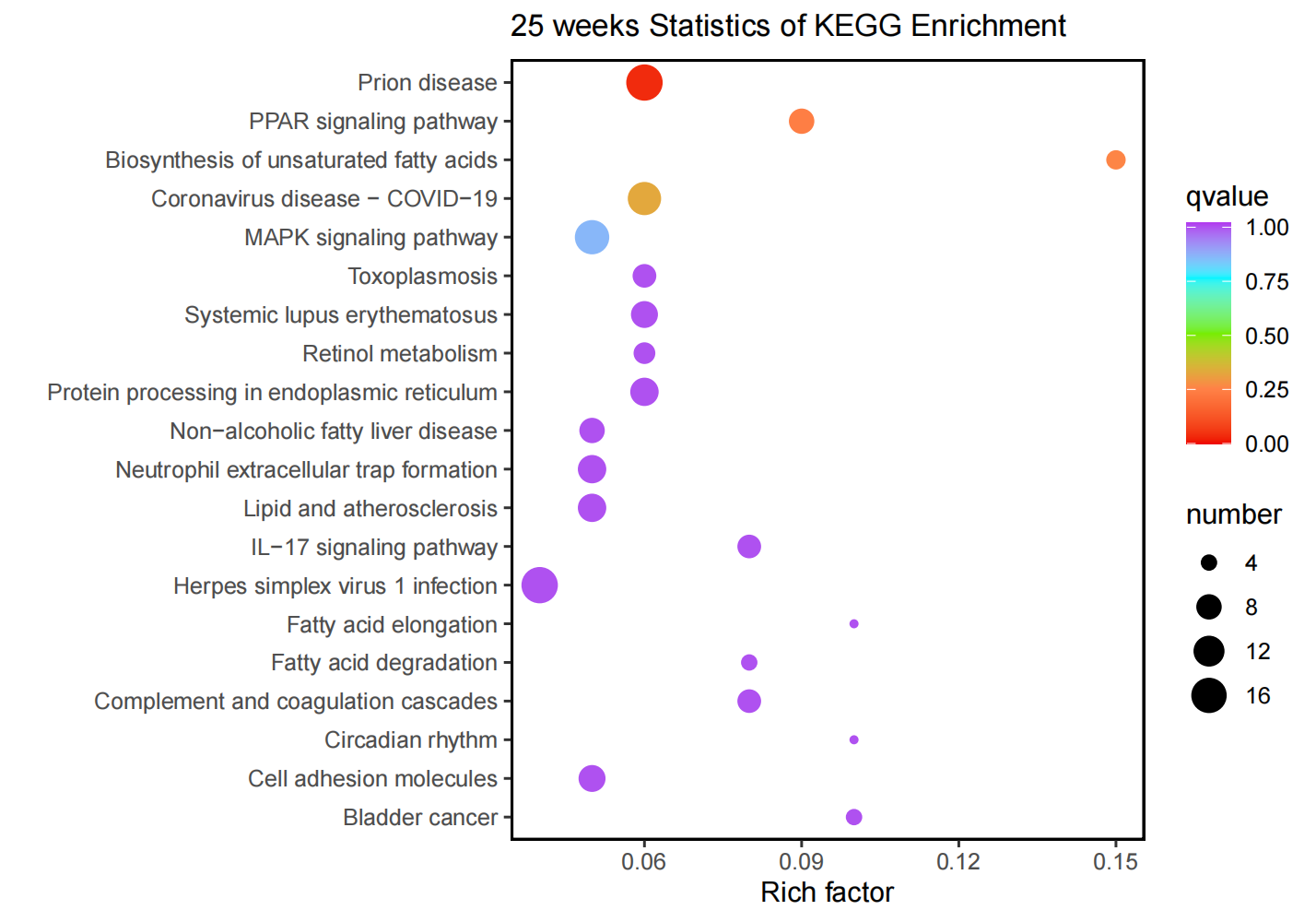

Supplement: Supplementary file 1 [file genes-16-00839-s001.zip › Figure S6.png]
